# Retrospektive Analyse des Schockraummanagements nichttraumatologisch kritisch kranker Kinder in einer universitären zentralen Notaufnahme (OBSERvE-DUS-PED-Studie)

**DOI:** 10.1007/s00101-024-01457-7

**Published:** 2024-09-02

**Authors:** Claudia Priebe, Hans Martin Bosse, Mark Michael, Olaf Picker, Michael Bernhard, Juliane Tautz

**Affiliations:** 1 Klinik für Allgemeine Pädiatrie, Kinderkardiologie und Neonatologie, Universitätsklinikum Düsseldorf, Heinrich-Heine-Universität, Moorenstraße 5, Düsseldorf, 40225 Deutschland; 2 Zentrale Notaufnahme, Universitätsklinikum Düsseldorf, Heinrich-Heine-Universität, Moorenstraße 5, 40225 Düsseldorf, Deutschland; 3 Klinik für Anästhesiologie, Universitätsklinikum Düsseldorf, Heinrich-Heine-Universität, Moorenstraße 5, 40225 Düsseldorf, Deutschland

**Keywords:** Kindernotfall, Schockraum, Distribution, Kritisch kranke Patienten, Versorgungskonzept, Pediatric emergency, Resuscitation room, Emergency department, Critically ill patients, Care concept

## Abstract

**Hintergrund:**

Die Etablierung eines nichttraumatologischen Schockraummanagements für kritisch kranke Kinder erscheint sinnvoll. In der vorliegenden Studie wurden Versorgungsdaten kritisch kranker nichttraumatologischer pädiatrischer Schockraumpatienten erhoben.

**Methoden:**

In der retrospektiven OBSERvE-DUS-PED-Studie (November 2019 bis Oktober 2022) wurden pädiatrische Patienten (Alter < 18 Jahre), die eine Schockraumversorgung aus nichttraumatologischer Ursache benötigten und der zentralen Notaufnahme zugeführt wurden, erfasst. Die routinemäßig dokumentierten Versorgungsdaten wurden gemäß dem OBSERvE-Datensatz dem Krankenhausinformationssystem MEDICO® und dem Patientendatenmanagementsystem COPRA® entnommen. Ein positives Ethikvotum der Medizinischen Fakultät der Heinrich-Heine-Universität lag vor (2023-2377).

**Ergebnisse:**

Für den 3‑jährigen Untersuchungszeitraum konnten 52 Schockraumpatienten evaluiert werden, wobei in der Kohorte Jugendliche zwischen 14 und 17 Jahren mit 37 % am häufigsten und Neugeborene/Säuglinge (0–1 Jahr) mit 8 % am seltensten vertreten waren. Die führenden Symptome, kategorisiert nach ABCDE-Problemen, waren Vigilanzminderung (D): 61 %, Herz-Kreislauf-Stillstand (C): 25 %, respiratorische Insuffizienz (B): 6 %, Atemwegsverlegung (A) und Umfeldfaktoren (E-Probleme) jeweils in 4 %. Prähospitale bzw. innerklinische Notfallmaßnahmen erfolgten in folgender Häufigkeit: peripherer (58 vs. 65 %), intraossärer (14 vs. 2 %) und zentraler Venenzugang (0 vs. 12 %), invasives Atemwegsmanagement (35 % vs. 8 %), kardiopulmonale Reanimation (21 vs. 10 %), Katecholamintherapie (15 vs. 17 %) und intraarterielle Druckmessung (0 vs. 17 %). Die mittlere Schockraumversorgungsdauer betrug 70 ± 43 min. Die 30-Tages-Letalität betrug 17 %.

**Schlussfolgerung:**

Die OBSERvE-DUS-PED-Studie zeigt die besonderen Herausforderungen nichttraumatologisch kritisch kranker Kinder in der prähospitalen und innerklinischen Versorgung. Die Vielfalt und Komplexität der Einweisungsdiagnosen sowie die unmittelbare vitale Bedrohung der Patienten lassen es sinnvoll erscheinen, derartige Patienten aufgrund der vorhandenen materiellen, infrastrukturellen und personellen Ressourcen in einer zentralen Notaufnahme primär zu behandeln.

**Zusatzmaterial online:**

Die Online-Version dieses Beitrags (10.1007/s00101-024-01457-7) enthält weitere Abbildungen und Tabellen.

## Abstract (en)

**Background:**

The establishment of a resuscitation room management for nontraumatic critically ill children appears to make sense. This study collected data of pediatric patients suffering from nontraumatic critically ill conditions treated in a resuscitation room.

**Methods:**

The retrospective OBSERvE-DUS-PED study (November 2019–October 2022) recorded pediatric patients (age < 18 years) who were admitted to the emergency department (ED) for resuscitation room care. The routinely documented data on treatment were taken from the hospital information system MEDICO® and the patient data management system COPRA® in accordance with the OBSERvE dataset. The study was approved by the Ethics Committee of the Medical Faculty of the Heinrich Heine University (2023-2377).

**Results:**

The study included 52 pediatric resuscitation room patients. Adolescents aged 14–17 years were the most frequent in the cohort representing 37% of the total and neonates/infants (0–1 year) were lowest at 8%. The most common symptoms categorized according to ABCDE problems were disturbance of consciousness (D) at 61%, cardiovascular failure (C) at 25%, respiratory insufficiency (B) at 6%, airway obstruction (A) and exposure/environment (E) problems each at 4%. The out-of-hospital and in-hospital emergency procedures were performed with the following frequencies: venous (58% vs. 65%), intraosseous (14% vs. 2%) and central venous access (0% vs. 12%), invasive airway management (35% vs. 8%), cardiopulmonary resuscitation (21% vs. 10%), vasopressors (15% vs. 17%), and intra-arterial pressure measurement (0% vs. 17%). The mean duration of resuscitation room management was 70 ± 43 min. The 30-day mortality was 17%.

**Conclusion:**

The OBSERvE-DUS-PED study demonstrates the major challenges in the care of critically ill nontraumatic pediatric patients, both in out-of-hospital and in-hospital management. The variety and complexity of the referral diagnoses as well as the immediate vital threat to the patients make it appear sensible to treat such patients primarily in a resuscitation room of the ED due to the available material, infrastructural and personnel resources.

## Einleitung

Schwer erkrankte und verletzte Kinder sind eine besonders vulnerable Patientengruppe in der Notfallmedizin. Nach der prähospitalen Versorgung im Rettungs- und Notarztdienst stehen für die Versorgung von Kindern bisher in Deutschland je nach Organisationsform und Klinikstruktur unterschiedliche Aufnahmelokalisationen im Krankenhaus zur Verfügung: Kindernotaufnahmen, Akutbehandlungsräume von Intensivstationen oder zentrale Notaufnahmen [19].

Als interdisziplinäre und integrative Nahtstelle zwischen der prähospitalen und frühen innerklinischen Versorgung stellt eine zentrale Notaufnahme eine wichtige Schlüssellokalisation dar. Für pädiatrische Traumapatienten ist die Versorgung in Schockräumen von Notaufnahmen durch die aktuelle S2K-Leitlinie „Polytraumaversorgung im Kindesalter“ ein etabliertes und akzeptiertes interdisziplinäres Konzept [8]. Für die Versorgung nichttraumatologisch kritisch kranker Kinder in zentralen Notaufnahmen besteht allerdings noch kein vergleichbar beschriebenes Versorgungskonzept. Nach der Etablierung eines nichttraumatologischen Schockraummanagements für kritisch kranke Erwachsene erscheint die Übertragung eines derartigen, interdisziplinär ausgerichteten Versorgungkonzeptes für nichttraumatologisch kritisch kranke Kinder sinnvoll [13]. Da Algorithmen, Medikamente und Vorgehensweisen aus der Erwachsenenmedizin sich nicht unmittelbar auf das Kindesalter übertragen lassen und bisher epidemiologische und versorgungstechnische Daten aus der Realversorgung für die Schockraumbehandlung nichttraumatisch kritisch kranker Kinder fehlen, war es Ziel der Studie, die Versorgung nichttraumatologisch kritisch kranker Kinder im Schockraummanagement einer universitären zentralen Notaufnahme zu analysieren, um erste Daten zu Epidemiologie, innerklinischen, therapeutischen und diagnostischen Maßnahmen und zum Behandlungsergebnis zu gewinnen. Entsprechende Erkenntnisse helfen, die erforderlichen Ressourcen für die Versorgung von Kindern im nichttraumatologischen Schockraum zu identifizieren, das Behandlungsergebnis abzuschätzen und die Notwendigkeit entsprechender Versorgungskonzepte im Vergleich zu erwachsenen Patienten abzugrenzen.

## Material und Methoden

### Studiendesign.

In der retrospektiven, monozentrischen Studie Observation of critically ill pediatric patients in the resuscitation room of the emergency department in Düsseldorf (OBSERvE-DUS-PED) wurden alle Kinder im Alter < 18 Jahren aus einem 3‑jährigen Untersuchungszeitraum (01.11.2019–30.10.2022) erfasst, die einer Schockraumversorgung in der Zentralen Notaufnahme des Universitätsklinikums Düsseldorf über den Rettungs- und Notarztdienst zugeführt wurden. Ein positives Ethikvotum der Medizinischen Fakultät der Heinrich-Heine-Universität lag vor (2023-2377).

### Studienzentrum.

Als interdisziplinäre Versorgungseinheit werden in der zentralen Notaufnahme traumatologisch und nichttraumatologisch kritisch kranke Kinder und Erwachsene behandelt. Parallel zur Kindernotaufnahme werden in der zentralen Notaufnahme des Universitätsklinikums jährlich über 45.000 Patienten behandelt, davon rund 10 % im Alter < 18 Jahren und 5 % der Kinder mit einem nichttraumatologischen Vorstellungsgrund [3].

**Abb. 1:**
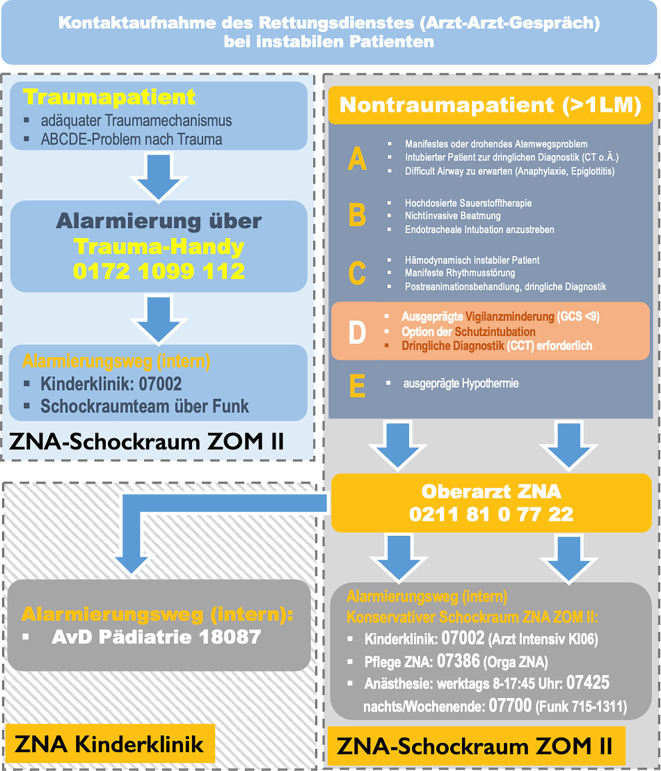
Standard Operating Procedure zu Übernahme und Distribution pädiatrischer Notfallpatienten am Universitätsklinikum Düsseldorf. Kinder mit relevantem Traumamechanismus kommen dabei zur Aufnahme in einen Traumaschockraum, kritisch kranke nichttraumatologische Kinder in den nichttraumatologischen Schockraum, und nichttraumatologische Kinder ohne Vitalgefährdung werden in die Kindernotaufnahme disponiert

Die Aufnahmelokalisation für kritisch kranke Kinder ist der Schockraum der zentralen Notaufnahme des universitären Maximalversorgers. Im Vorfeld der Untersuchung erfolgte eine multidisziplinäre und interprofessionelle Abstimmung des innerklinischen Versorgungskonzeptes kritisch kranker nichttraumatologischer Kinder mit allen beteiligten Fachdisziplinen: Das interdisziplinäre Schockraummanagement kritisch kranker Kinder erfolgt in einem multidisziplinären Team, bestehend aus ärztlichen und pflegerischen Mitarbeitenden der Anästhesiologie, Pädiatrie, Radiologie und der klinischen Akut- und Notfallmedizin. Bei einer sekundär identifizierten chirurgischen Ursache des Erkrankungszustandes wird das Behandlungsteam durch unfall-, allgemein-, kinder- und neurochirurgische Fachdisziplinen ergänzt. Eine spezifische Anmeldeprozedur wurde mit den Partnern des Rettungs- und Notarztdienstes der Region vereinbart (Abb. 1). Der wesentliche Argumentationsgrund für das Versorgungkonzept nichttraumatologisch kritisch kranker Kinder im Schockraum war die Verfügbarkeit aller notwendigen materiellen, infrastrukturellen und personellen Ressourcen, um dieses vulnerable Patientenkollektiv mit höchstmöglicher Expertise adäquat und insbesondere zeitgerecht zu behandeln.

### Datenerfassung und Definitionen.

Die routinemäßig dokumentierten Versorgungsdaten wurden unter Nutzung des OBSERvE-Datensatzes [5, 9, 18] dem Krankenhausinformationssystem MEDICO® (Fa. Cerner Deutschland GmbH, Itstein, Deutschland) und dem Patientendatenmanagementsystem COPRA® (Fa. COPRA System GmbH, Berlin, Deutschland) entnommen und in eine elektronische Datenbank überführt (Microsoft Excel, Version 16.37, Fa. Microsoft Corporation, Redmond, WA, USA). Die deskriptive Analyse erfolgte dann anhand des anonymisierten Datensatzes. Die Anforderungen an den Datenschutz nach der Datenschutzgrundverordnung (DSGVO) wurden eingehalten. Die Patientenkohorte wurde in 5 Altersgruppen unterteilt: Neugeborene und Säuglinge (0 bis 11 Monate), Klein- (1 bis 4 Jahre) und Schulkinder (5 bis 8 Jahre), Präpubertäre (9 bis 13 Jahre) und Jugendliche (14 bis 17 Jahre).

### Ein- und Ausschlusskriterien.

In die Auswertung wurden Kinder mit einer hochgradigen Beeinträchtigung der Vitalfunktionen aufgrund einer nichttraumatologisch Ursache eingeschlossen und die führende Symptomatik nach dem ABCDE-Schema klassifiziert. Von der Analyse ausgeschlossen wurden Kinder mit einem primären Traumamechanismus bzw. diejenigen Kinder, die nach einem Dispositionstelefonat mit dem oberärztlichen Koordinator der zentralen Notaufnahme als nicht kritisch krank eingeschätzt wurden.

### Prähospitale und Versorgung im Schockraum.

Aus den Protokollen des Rettungs- und Notarztdienstes wurden die prähospital vorliegenden ABCDE-Probleme, der NACA-Score (Zusatzmaterial online: Tab. S1) und der Summen-Score der Glasgow Coma Scale (GCS) dokumentiert und die durchgeführten Notfallmaßnahmen erfasst. Für die Schockraumversorgung wurden die Vitalparameter bei Aufnahme, die Verdachtsdiagnose, die diagnostischen und therapeutischen Maßnahmen, die Schockraumverweildauer und das Behandlungsergebnis dokumentiert.

### Statistik.

Zur statistischen Auswertung wurden die Kinder in vorab definierten Altersgruppen bezüglich unterschiedlicher Merkmale miteinander verglichen. Alle Daten wurden mittels Excel (Microsoft Excel, Version 16.37, Fa. Microsoft Corporation, Redmond, WA, USA) analysiert und die Abbildungen über DataGraph (Version 4.6.1, Fa. Visual Data Tool Inc., Chapel Hill, NC, USA) sowie das Open Source Programm „SankeyMATIC“ (https://sankeymatic.com/build/) erstellt. Für qualitative Merkmale wurden relative und absolute Häufigkeiten prozentual angegeben. Mithilfe des Chi^2^-Tests wurden qualitative Unterschiede zwischen einzelnen Patientenkollektiven untersucht. Quantitative Variablen wurden als Mittelwert ± Standardabweichung (SD) dargestellt und Minimum, Maximum sowie Median ermittelt. Der *t*-Test wurde bei quantitativen, annähernd normalverteilten Merkmalen für 2 unverbundene Stichproben verwendet. Bei schief verteilten Variablen wurde dagegen der U‑Test von Mann und Whitney angewandt.

## Ergebnisse

Im 3‑jährigen Untersuchungszeitraum wurden insgesamt 52 nichttraumatologisch kritisch kranke Kinder im Schockraum erfasst (0,04 % aller Patientenkontakte der zentralen Notaufnahme während des 3‑jährigen Untersuchungszeitraums).

### Patientencharakteristika

In der Patientenkohorte waren am häufigsten Jugendliche und am seltensten Neugeborene und Säuglinge vertreten (Tab. 1). Der Anteil weiblicher Patienten lag bei 46,2 %. Hinsichtlich der Erkrankungsschwere nach notärztlicher Einschätzung kamen die meisten Kinder unter nichtauszuschließender oder akuter Lebensgefahr und in Reanimationsbereitschaft (NACA IV–V; 75,0 %) oder nach bzw. unter Reanimationsmaßnahmen (NACA VI; 15,5 %) zur Aufnahme (Tab. 1).

**Tab. 1:** Patientencharakteristika und prähospitale Versorgung der nichttraumatologischen kritisch kranken Kinder

Altersgruppen	0 bis 12 Monate	1 bis 4 Jahre	5 bis 8 Jahre	9 bis 13 Jahre	14 bis 17 Jahre	Gesamtgruppe
*n* (%)	4 (7,7)	13 (25,0)	7 (13,5)	9 (17,3)	19 (36,5)	52 (100,0)
m:w (*n*)	0:4	11:2	4:3	4:5	9:10	28:24
**Erkrankungsschwere, NACA (*n*, (%))**						
III	0	0	0	1 (1,9)	3 (5,8)	4 (7,7)
IV	0	6 (11,6)	3 (5,8)	2 (3,9)	7 (13,5)	18 (34,6)
V	2 (3,9)	6 (11,6)	3 (5,8)	5 (9,6)	5 (9,6)	21 (40,4)
VI	2 (3,9)	1 (1,9)	1 (1,9)	1 (1,9)	3 (5,8)	8 (15,4)
k. A.	0	0	0	0	1 (1,9)	1 (1,9)
**Führendes ABCDE-Problem (*n*, (%))**						
A	0	1 (1,9)	0	1 (1,9)	0	2 (3,9)
B	0	0	0	0	3 (5,8)	3 (5,8)
C	3 (5,8)	2 (3,9)	1 (1,9)	3 (5,8)	4 (7,7)	13 (25,0)
D	1 (1,9)	10 (19,2)	6 (11,6)	4 (7,7)	11 (21,2)	32 (61,5)
E	0	0	0	1 (1,9)	1 (1,9)	2 (3,9)
**Prähospitale Versorgung (*n*, (%))**						
*Atemwegsmanagement*						
Sauerstoffgabe	0	6 (11,6)	0	2 (3,9)	2 (3,9)	10 (19,2)
Maskenbeatmung	1 (1,9)	2 (3,9)	0	0	0	3 (5,8)
ETI	2 (3,9)	5 (9,6)	3 (5,8)	2 (3,9)	7 (13,5)	19 (36,5)
Kapnographie bei ETI	2 (3,9)	5 (9,6)	3 (5,8)	2 (3,9)	7 (13,5)	19 (36,5)
CPAP bei TS	0	0	0	1 (1,9)	1 (1,9)	2 (3,9)
*Venenzugangswege*						
Peripher	1 (1,9)	7 (13,5)	3 (5,8)	6 (11,6)	14 (26,9)	31 (59,6)
Intraossärer	2 (3,9)	1 (1,9)	1 (1,9)	2 (3,9)	1 (1,9)	7 (13,5)
Kein	1 (1,9)	5 (9,6)	4 (7,7)	1 (1,9)	4 (7,7)	14 (26,9)
**Kardiopulmonale Reanimation (*n*, (%))**						
CPR	3 (5,8)	2 (3,9)	1 (1,9)	2 (3,9)	3 (5,8)	11 (21,2)
Beobachteter HKS	1 (1,9)	0	1 (1,9)	2 (3,9)	3 (5,8)	7 (13,5)
Laienreanimation	2 (3,9)	2 (3,9)	0	1 (1,9)	1 (1,9)	6 (11,6)
Telefonreanimation	1 (1,9)	1 (1,9)	0	0	0	2 (3,9)
**Mittlere Reanimationsdauer [MW** **±** **SD, min]**						20 ± 24
**Erste Rhythmusanalyse durch RD (*n*, (%))**						
Asystolie	1 (1,9)	1 (1,9)	1 (1,9)	0	0	3 (5,8)
PEA	0	0	0	0	1 (1,9)	1 (1,9)
pVT	0	0	0	1 (1,9)	1 (1,9)	2 (3,9)
KF	0	0	0	0	1 (1,9)	1 (1,9)
Sinusrhythmus	2 (3,9)	1 (1,9)	0	1 (1,9)	0	4 (7,7)
Defibrillation	0	0	0	1 (1,9)	2 (3,9)	3 (5,8)
ACCD	0	0	0	0	1 (1,9)	1 (1,9)
Katecholamine	1 (1,9)	1 (1,9)	1 (1,9)	0	3 (5,8)	6 (11,6)
30:2 KVV	0	0	0	2 (3,9)	0	2 (3,9)
15:2 KVV	2 (3,9)	1 (1,9)	0	0	0	3 (5,8)
Kontinuierlich	0	1 (1,9)	1 (1,9)	0	3 (5,8)	5 (9,6)
Unklares Verhältnis	1 (1,9)	0	0	0	0	1 (1,9)
**ROSC vor Ankunft RD**	2 (3,9)	1 (1,9)	0	1 (1,9)	0	4 (7,7)

*M* männlich, *w* weiblich, *NACA* National Advisory Committee of Aeronautics, *k.* *A.* keine Angaben, *ETI* endotracheale Intubation, *CPAP* „continous positive airway pressure“, *CPR* kardiopulmonale Reanimation, *TS* bereits einliegendes Tracheostoma, *HKS* Herz-Kreislauf-Stillstand, *ROSC* „resuscitation of spontaneous circulation“, *PEA* pulslose elektrische Aktivität, *pVT* pulslose ventrikuläre Tachykardie, *ACCD* „automated chest compression device“, *KF* Kammerflimmern, *MW* Mittelwert, *SD* Standardabweichung, *RD* Rettungs- und Notarztdienst, *KVV* Kompression-Ventilation-Verhältnis

### Führende ABCDE-Probleme

**Abb. 2:**
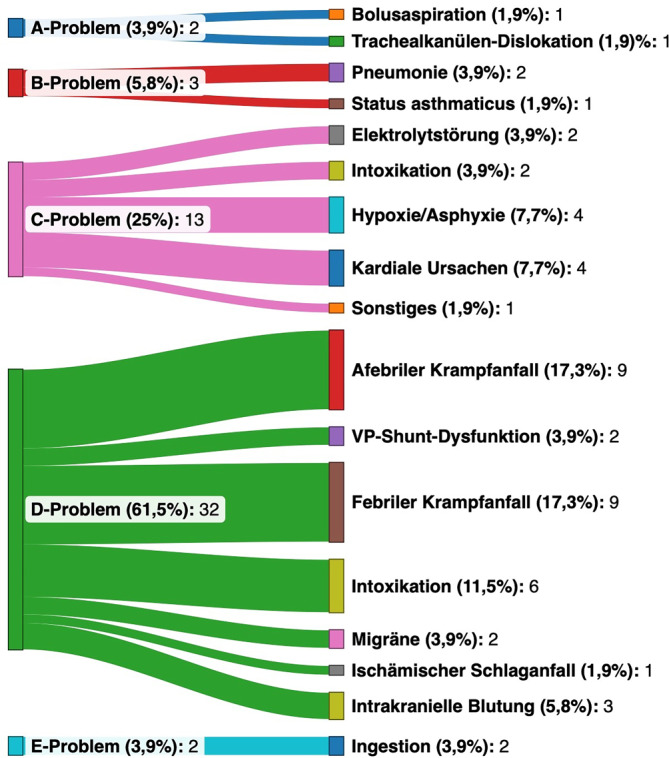
Führende ABCDE-Probleme und zugrunde liegende Erkrankungen bei den 52 nichttraumatologisch kritisch kranken Kindern

Der überwiegende Anteil der Kinder kam mit einem führenden D‑Problem in den Schockraum, in der absteigenden Reihung gefolgt von C‑, B‑ und A‑/E-Problemen (Tab. 1). Deren zugrunde liegende Ursachen und Erkrankungen sind in Abb. 2 dargestellt. A‑Probleme waren durch Atemwegsverlegungen (Bolusaspiration, Dislokation einer Trachealkanüle bei Heimbeatmung) verursacht. Ein Status asthmaticus und ambulant erworbene Pneumonien führten zu den B‑Problemen. C‑Probleme wurden durch Intoxikationen, Elektrolytstörungen, kardiale Grunderkrankungen, supraventrikuläre Tachykardien, schwere Hypoxie/Asphyxie, eine primäre Nebennierenrindeninsuffizienz (Autoimmunadrenalitis) bzw. ein „apparent life threatening event“ (ALTE) verursacht. Bei 10 der 13 Patienten (77 %) mit führendem C‑Problem bei Schockraumaufnahme kam es prähospital zu einer Reanimationssituation. Als Ursachen der D‑Probleme fanden sich afebrile Krampfanfälle, ischämische Schlaganfälle, intrakranielle Blutungen, Shunt-Dysfunktionen, Migräne, Intoxikationen oder sonstige Ursachen. Alle E‑Probleme entfielen auf Intoxikationen. Eine Übersicht über die Verteilung der den ABCD-Problemen zugrunde liegenden Erkrankungen und den jeweiligen Altersgruppen bieten die Abb. S1 und S2 im Zusatzmaterial online.

### Prähospitale Versorgung

#### Atemwegsmanagement und Beatmung.

Spontan atmend mit Sauerstoffapplikation über eine Gesichtsmaske wurden 19,0 % der Kinder zugeführt (Tab. 1). Prähospital kamen eine alleinige Maske-Beutel-Beatmung bis zur Schockraumaufnahme selten und Luftbrücken (Guedel‑/Wendl-Tubus) bzw. supraglottische Atemwegshilfsmittel nicht zur Anwendung. Eine invasive Atemwegssicherung fand in 36,5 % der Fälle mittels endotrachealer Intubation unter regelhafter Kapnometrie statt (Tab. 1). Eine nichtinvasive Atemunterstützung mittels Sauerstoffapplikation kam in 2 Fällen bei bereits einliegendem Tracheostoma zur Anwendung.

#### Etablierung von Gefäßzugängen.

Prähospital waren in mehr als der Hälfte der Fälle ein peripherer Venenzugang, in 13,5 % ein intraossärer und in 26,9 % kein Zugang etabliert worden.

#### Prähospitale Reanimationsmaßnahmen.

Bei 21,2 % der nichttraumatologisch kritisch kranken Kinder waren prähospitale Reanimationsmaßnahmen notwendig (Tab. 1). Die Mehrheit der Herz-Kreislauf-Stillstände trat beobachtet ein; bei der Hälfte der Fälle erfolgte eine Laienreanimation, und bei jedem 5. reanimationspflichtigen Kind fand eine Telefonreanimation statt. Nichtschockbare und schockbare Rhythmen lagen im Verhältnis 3:4 vor. Alle Patienten mit schockbarem Initialrhythmus waren ≥ 13 Jahre und alle Patienten mit initialer Asystolie oder pulsloser elektrischer Aktivität ≤ 8 Jahre alt. Bei allen schockbaren Initialrhythmen wurde prähospital eine Defibrillation durchgeführt. Auf ein „automated chest compression device“ (ACCD) wurde bei einem Patienten zurückgegriffen. Prähospital erhielt die Hälfte der reanimationspflichtigen Kinder Katecholamingaben. Bei einem Drittel der Patienten nach Laienreanimation lag bei Eintreffen des Rettungsdienstes ein Sinusrhythmus mit suffizientem Spontankreislauf vor. Die Reanimationsdauer lag im Median bei 7 min (Min–Max: 2–60, MW ± SD: 20 ± 24 min).

### Schockraumversorgung

**Abb. 3:**
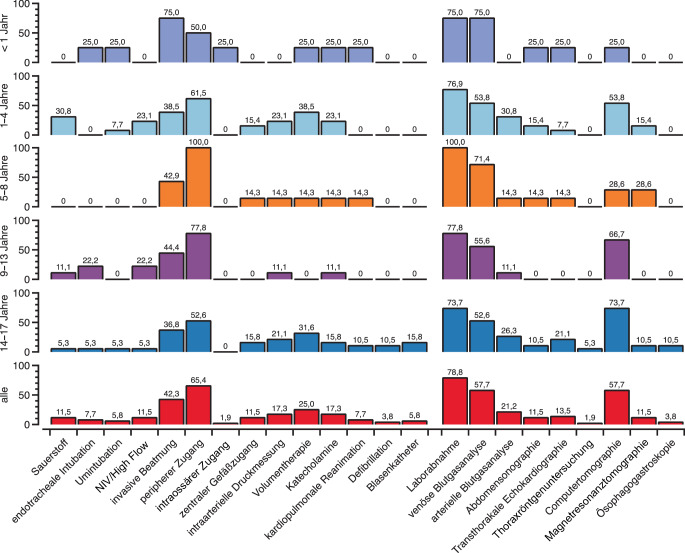
Therapeutische (*links*) und diagnostische Maßnahmen (*rechts*) während des Schockraumversorgung bei den 52 nichttraumatologisch kritisch kranken Kindern. Angaben in Prozent in der jeweiligen Altersgruppe

Von den kritisch kranken Kindern hatten 60 % bei Schockraumaufnahme einen Summen-Score auf der GCS < 9 Punkte (Tab. 2). In über jeweils 10 % lag eine Hypothermie bzw. Fieber vor. Eine Hypoxie lag in 7,7 % und eine altersadjustierte Hypotension in 13,5 % der Fälle vor (Tab. 2). Eine Übersicht über die im Schockraum eingesetzten Notfallmaßnahmen bietet Abb. 3.

**Tab. 2:** Schockraumversorgung

Altersgruppen	0 bis 11 Monate	1 bis 4 Jahre	5 bis 8 Jahre	9 bis 13 Jahre	14 bis 17 Jahre	Gesamtgruppe
*n* (%)	4 (7,7)	13 (25,0)	7 (13,5)	9 (17,3)	19 (36,5)	52 (100,0)
**Glasgow-Koma-Skala (*n*, (%))**						
13–15	0	3 (5,8)	1 (1,9)	3 (5,8)	7 (13,5)	14 (26,9)
9–12	1 (1,9)	0	1 (1,9)	0	2 (3,9)	4 (7,7)
≤ 8	3 (5,8)	10 (19,2)	4 (7,7)	4 (7,7)	9 (17,3)	30 (57,7)
k. A.	0	0	1 (1,9)	2 (3,9)	1 (1,9)	4 (7,7)
**Körpertemperatur (°C) (*n*, (%))**						
≤ 35,9	2 (3,9)	2 (3,9)	1 (1,9)	0	2 (3,9)	7 (13,5)
36,0–38,0	0	6 (11,5)	1 (1,9)	6 (11,5)	10 (19,2)	23 (44,2)
≥ 38,1	0	2 (3,9)	3 (5,8)	1 (1,9)	0	6 (11,5)
k. A.	2 (3,9)	3 (5,8)	2 (3,9)	2 (3,9)	7 (13,5)	16 (30,8)
**Blutdruck, systolisch (mm** **Hg) (*n*, (%))**						
> 80	1 (1,9)	8 (15,4)	5 (9,6)	9 (17,3)	17 (32,7)	40 (75)
< 80	1 (1,9)	2 (3,9)	2 (3,9)	0	2 (3,9)	7 (13,5)
k. A.	2 (3,9)	3 (5,8)	0	0	0	5 (9,6)
Hypotension	0	1 (1,9)	2 (3,9)	1 (1,9)	3 (5,8)	7 (13,5)
**Blutgasanalyse (pH-Wert) (*n*, (%))**						
> 7,25	2 (3,9)	6 (11,5)	3 (5,8)	4 (7,7)	8 (15,4)	23 (44,2)
< 7,25	0	1 (1,9)	1 (1,9)	1 (1,9)	3 (5,8)	6 (11,5)
< 7,00	1 (1,9)	1 (1,9)	1 (1,9)	1 (1,9)	2 (3,9)	6 (11,5)
k. A.	1 (1,9)	5 (9,6)	2 (3,9)	3 (5,8)	6 (11,5)	17 (32,7)
**Herzfrequenz (bpm) (*n*, (%))**						
Normofrequent	1 (1,9)	7 (13,5)	2 (3,9)	5 (9,6)	12 (23,1)	27 (51,9)
Tachykard	3 (5,8)	5 (9,6)	4 (7,7)	3 (5,8)	8 (15,4)	23 (44,2)
Bradykard	0	0	1 (1,9)	0	0	1 (1,9)
k. A.	0	1 (1,9)	0	0	0	1 (1,9)
**Pulsoxymetrisch gemessene Sauerstoffsättigung (%) (*n*, (%))**						
> 90 %	2 (3,9)	13 (25)	6 (11,5)	7 (13,5)	19 (36,5)	47 (90,4)
< 90 %	2 (3,9)	0	1 (1,9)	2 (3,9)	0	5 (9,6)

Normwerte systolischer Blutdruck: 0 bis 11 Monate: > 50–70 mm Hg; 1 bis 4 Jahre: > 70–80 mm Hg; 5 bis 8 Jahre: > 80–85 mm Hg; > 9 Jahre: > 90 mm Hg

Normwerte Herzfrequenz (bpm = beats per minute): 0 bis 11 Monate: 100–160 bpm; 1 bis 4 Jahre: 80–130 bpm; 5 bis 8 Jahre: 70–110 bpm; 9 bis 13 Jahre: > 60–100 bpm; > 14 Jahre: 50–100 bpm

*k.* *A.* keine Angaben

#### Atemwegsmanagement und Beatmung.

Bei 7,7 % der Kinder wurde eine endotracheale Intubation im Schockraum durchgeführt. Eine Umintubation (2-malig größerer, einmal kleinerer Tubusdurchmesser) im Schockraum war bei 15,8 % der prähospital intubierten Patienten zur Beatmungsoptimierung notwendig. Am Ende der Schockraumversorgung waren 53,8 % der Kinder nichtinvasiv oder invasiv beatmet.

#### Etablierung von Gefäßzugängen.

Einen peripheren Venenzugang erhielten 65,3 %, einen zentralen Gefäßzugang 11,5 % und einen intraossären Zugang 1,9 % der Kinder. Bei 17,3 % der Kinder wurde eine arterielle Druckmessung etabliert. Eine Volumentherapie mit kristalloider Infusionslösung wurde bei einem Viertel der Patienten durchgeführt. Eine Bluttransfusion erhielten 4 % der Kinder im Schockraum.

#### Diagnostische Verfahren.

Eine Übersicht über die eingesetzten diagnostischen Verfahren bietet Tab. 2. Am häufigsten wurden bildgebende Großgeräte in 69,2 % eingesetzt, davon mittels Computertomographie (CT) in 83,3 % und Magnetresonanztomographie (MRT) in 16,7 % der Fälle. Eine Thoraxröntgenuntersuchung erhielten 1,9 %, eine Abdomensonographie 11,5 % und eine transthorakale Echokardiographie 13,5 % der Kinder. Eine Labordiagnostik, inklusive Blutgasanalyse, erfolgte in 78,8 % und die Abnahme von Blutkulturen in 3,8 %. Ein Blasenkatheter wurde bei 5,8 % der Kinder verwendet.

#### Innerklinische Reanimationsmaßnahmen.

Bei Aufnahme im Schockraum hatten 81,8 % der 11 prähospital reanimierten und dem Krankenhaus zugeführten Kinder eine spontane Kreislauffunktion, bei 2 Patienten erfolgte die Zuführung unter Reanimationsmaßnahmen. Bei 2 der prähospital reanimierten Kinder musste die kardiopulmonale Reanimation während der Schockraumversorgung wieder aufgenommen werden. Bei 17,3 % der Patienten wurden im Schockraum zur Kreislaufstabilisierung bzw. zur Reanimationsbehandlung Katecholamine eingesetzt. Eine Defibrillation musste bei 3,8 % der Patienten durchgeführt werden. Ein Patient wurde mittels ACCD reanimiert.

#### Versorgungsdauer, Verlegungslokalisation und Behandlungsergebnis.

Die mittlere Versorgungsdauer im Schockraum betrug 70 ± 43 min (Min–Max: 0–186, Median 61,5 min, Zeiten inkl. einer sich anschließenden Großgerätediagnostik). Kinder mit einer prähospitalen Reanimationssituation wiesen im Vergleich zu Kindern ohne ein Reanimationsereignis keine längere Versorgungsphase im Schockraum auf (88 ± 42 vs. 65 ± 42 min, *p* = 0,113). Die meisten Kinder wurden auf die pädiatrische Intensivstation oder Normalstation aufgenommen (Tab. 3). Die Schockraumletalität, die 24-h- bzw. 30-Tages-Letalität betrugen 1,9 %, 11,5 % bzw. 17,3 %. Kinder mit einem prähospitalen Reanimationsereignis wiesen eine 30-Tages-Letalität von 63,4 % und ohne ein entsprechendes Ereignis von 4,9 % auf (Tab. 3).

**Tab. 3:** Behandlungsergebnis und Verlegungslokalisation

Altersgruppen	0 bis 11 Monate	1 bis 4 Jahre	5 bis 8 Jahre	9 bis 13 Jahre	14 bis 17 Jahre	Gesamtgruppe
*n* (%)	4 (7,7)	13 (25,0)	7 (13,5)	9 (17,3)	19 (36,5)	52 (100,0)
**Behandlungsergebnis (*n*, (%))**						
24-h-Letalität	1 (1,9)	0	1 (1,9)	0	4 (7,7)	6 (11,5)
30-Tages-Letalität	2 (3,9)	1 (1,9)	1 (1,9)	0	5 (9,6)	9 (17,3)
*Krankenhausletalität von Patienten …*						
mit prähospitaler Reanimation	2 (3,9)	1 (1,9)	1 (1,9)	0	3 (5,8)	7/11 (63,4)
ohne prähospitale Reanimation	0	0	0	0	2 (3,9)	2/41 (4,9)
**Verlegungslokalisation nach Schockraumversorgung (*n*, (%))**						
ICU, pädiatrisch	2 (3,9)	10 (19,2)	4 (7,7)	6 (11,5)	11 (21,2)	33 (63,5)
ICU, „adult“	0	0	0	0	2 (3,9)	2 (3,9)
Operative Intervention	0	0	0	0	1 (1,9)	1 (1,9)
Ambulante Versorgung	0	0	0	1 (1,9)	0	1 (1,9)
Normalstation	1 (1,9)	3 (5,8)	3 (5,8)	2 (3,9)	5 (9,6)	14 (26,9)
Exitus letalis, Schockraum	1 (1,9)	0	0	0	0	1 (1,9)

*k.* *A.* keine Angaben, *ICU* Intensivstation

## Diskussion

In der vorliegenden retrospektiven, monozentrischen OBSERvE-DUS-PED-Studie wurde die Schockraumversorgung von 52 kritisch kranken nichttraumatologischen Kindern in einer universitären zentralen Notaufnahme analysiert. Die Datenanalyse lieferte erste Erkenntnisse zu Epidemiologie, den prähospitalen und innerklinischen diagnostischen und therapeutischen Notfallmaßnahmen und zum Behandlungsergebnis von kritisch kranken nichttraumatologischen Kindern, die durch ein multidisziplinäres Schockraumteam versorgt wurden.

### Aufnahmelokalisation und Versorgungskonzepte.

In der klinischen Praxis existiert derzeit kein einheitliches Versorgungskonzept für die Aufnahme von kritisch kranken nichttraumatologischen Kindern, dementsprechend weisen die bisherigen Strategien eine große Diversität auf [19]: Die initiale Aufnahme kritisch kranker Kinder erfolgt sowohl in Kindernotaufnahmen als auch direkt auf Kinderintensivstationen oder in den Schockräumen von zentralen Notaufnahmen, mit und ohne angrenzende Kinderklinik. Weltweit werden die meisten Kinder in allgemeinen zentralen Notaufnahmen und nicht in spezialisierten pädiatrischen Notaufnahmen behandelt [4]. Welches das optimale Versorgungskonzept ist, kann zum jetzigen Zeitpunkt noch nicht abschließend wissenschaftlich beantwortet werden und bleibt bislang eine Entscheidung, die im Wesentlichen auf gewachsenen lokalen Strukturen beruht.

Die Aufnahmelokalisation für das Patientenkollektiv in der OBSERvE-DUS-PED-Studie und damit wesentlicher Studienfokus ist der Schockraum der zentralen Notaufnahme eines universitären Maximalversorgers. Im Vorfeld hat die Professionalisierung der akut- und notfallmedizinischen Versorgung in der zentralen Notaufnahme am eigenen Standort zu einer multidisziplinären und interprofessionellen Abstimmung des innerklinischen Versorgungskonzeptes kritisch kranker nichttraumatologischer Kinder mit allen beteiligten Fachdisziplinen geführt: In einem multiprofessionellen Teamansatz, bestehend aus ärztlichen und pflegerischen Mitarbeitenden der Anästhesiologie, Pädiatrie und klinische Akut- und Notfallmedizin sowie, je nach Krankheitsentität, auch Mitarbeitenden aus radiologischen und chirurgischen Fachdisziplinen, werden Kinder direkt in den Schockraum der zentralen Notaufnahme aufgenommen. Hierzu wurde eine spezifische Anmeldeprozedur mit den Partnern des Rettungs- und Notarztdienstes der Region vereinbart (Abb. 1). Der wesentliche Argumentationsgrund für das Versorgungkonzept nichttraumatologisch kritisch kranker Kinder im Schockraum war die Verfügbarkeit aller notwendigen materiellen, infrastrukturellen und personellen Ressourcen, um dieses vulnerable Patientenkollektiv mit höchstmöglicher Expertise adäquat und insbesondere zeitgerecht zu behandeln.

### Datenanalyse und Vorarbeiten.

Der methodische Ansatz und die Grundlage des erhobenen Datensatzes der OBSERvE-DUS-PED-Studie waren vorangegangene prospektive und retrospektive Untersuchungen zur Versorgung nichttraumatologischer erwachsener Patienten in den Schockräumen zweier universitärer zentraler Notaufnahmen [5, 9, 18]. Besonderer Fokus lag in der OBSERvE-DUS-PED-Studie auf dem transsektoralen Forschungsansatz an der Nahtstelle zwischen prähospitaler und innerklinischer Versorgung für kritisch kranke nichttraumatologische Kinder. Vergleichbar mit der EpiZNA-Studie [3] wurde in unterschiedlichen (inter‑)nationalen prähospitalen Studien der pädiatrische Anteil am Einsatzspektrum mit über 10 % beschrieben [6, 11, 12], davon übereinstimmend 5 % mit einem nichttraumatologischen Vorstellungsgrund [3, 31]. Auf kritisch kranke, nichttraumatologische Kinder im Schockraum entfiel mit 0,04 % aber nur ein geringer Anteil aller Notaufnahmepatienten im Studienzeitraum. Auch andere Untersuchungen bestätigen, dass der tatsächliche Anteil kritisch kranker oder schwer verletzter Kinder am Gesamtpatientenspektrum einer Notaufnahme gering ist: Lutz et al. [21] fanden am Universitätsklinikum in Lausanne einen Anteil von 0,46 % für traumatologische und nichttraumatologische kritisch kranke Kinder. Im Vergleich dazu wurde für erwachsene kritisch kranke nichttraumatologische Schockraumpatienten mit bis zu 1,5 % ein deutlicher höherer Anteil am Notaufnahmekollektiv ermittelt [18].

### Epidemiologische Daten.

Das Patientenkollektiv der OBSERvE-DUS-PED-Studie setzte sich zu einem Drittel aus Jugendlichen und einem Drittel aus Klein- und Schulkindern zusammen. Im Einklang mit anderen Studienergebnissen [11, 14, 24] war der Anteil der besonders vulnerablen Altersgruppe der Neugeborenen und Säuglinge (< 1 Jahr) im Studienzeitraum anteilsmäßig am geringsten. Die übrige Verteilung steht im Kontrast zu mehreren Studien, in denen die Gruppe der Kleinkinder (1 bis 5 Jahre) den größten Anteil einnahm [6, 10, 11, 15]. Mögliche Ursachen für die hier vorliegende Diskrepanz sind unterschiedliche Altersdefinitionen und (teilweise) der Ausschluss Jugendlicher ab 14 Jahren in einigen Untersuchungen [6, 10, 11, 15]. Ein weiterer bedeutender Selektionsbias liegt im Einschlusskriterium des kritisch kranken, nichttraumatologischen Erkrankungszustandes der Kinder in die OBSERvE-DUS-PED-Studie vor dem Hintergrund der vorab definierten Dispositionsstrategie vor (Abb. 1), während in anderen Untersuchungen das gesamte Spektrum der Patientenkontakte pädiatrischer Notaufnahmen erfasst wurde und damit unabhängig vom Gesundheitszustand des Kindes bzw. vom dokumentierten NACA-Score ist. Daher lag in der OBSERvE-DUS-PED-Studie bei > 90 % der Kinder eine nichtauszuschließende oder akute Lebensgefahr vor, oder die Zuführung erfolgte in Reanimationsbereitschaft oder nach bzw. unter Reanimationsmaßnahmen (NACA IV–VI). Dieser hohe Anteil an Patienten mit NACA IV–VI bestätigt die erfolgreiche Umsetzung der adressierten Dispositionsstrategie kindlicher Notfälle mit angenommener akuter Vitalgefährdung in den Schockraum der zentralen Notaufnahme am eigenen Standort. Im bodengebundenen Rettungs- und Notarztdienst bzw. in der Luftrettung wurde ein sehr variabler Anteil der NACA-V- und NACA-VI-Patienten von 20–84 % beschrieben, jedoch wurden in diese Studien auch Kinder mit Traumafolge eingeschlossen [14, 21]. Mehrere Studien belegen, dass in unselektionierten Patientenkollektiven Traumata einen relevanten und mit steigendem Kindesalter auch mehrheitsbildenden Anteil ausmachen [6, 11, 14, 21, 24].

### Nichttraumatologisches Erkrankungsspektrum.

Als führende Problematik mit über 60 % zeigte sich in der OBSERvE-DUS-PED-Studie das D‑Problem in Übereinstimmung mit der Literaturdatenlage; die führende, zugrunde liegende Aufnahmediagnose war der (febrile bzw. afebrile) Krampfanfall mit einer persistierenden Bewusstseinsstörung [10–12, 24]. Im Unterschied zur OBSERvE-DUS-PED-Studie wurden in anderen Untersuchungen respiratorische Notfälle als führende nichttraumatologische Behandlungsursachen beschrieben (z. B. Pneumonien bzw. Aspirationen im Säuglingsalter, stenosierende Laryngotracheitis im Kleinkindalter, Hyperventilation und Asthma bronchiale im Schulkind- und Jugendalter), und erst als zweithäufigste Ursache der zumeist infektiös getriggerte, febrile Krampfanfall angeführt [6, 14, 15, 25, 28–30]. Erwachsene, nichttraumatologische Schockraumpatienten wiesen in korrespondierenden Untersuchungen am gleichen Standort auch D‑Probleme als führendes Problem auf [18]. Im Allgemeinen machen neurologische Notfälle in der Kindernotaufnahme rund ein Drittel der Fälle mit Vorliegen einer hochgradigen akuten Störung aus [26]. Etwa 75 % der Kinder mit akuten neurologischen Symptomen weisen Krampfanfälle, Kopfschmerzen oder andere paroxysmale Ereignisse auf; lebensbedrohliche Zustände betreffen dabei nur einen geringen Anteil der Patienten [26].

In der OBSERvE-DUS-PED-Studie wurden 25 % der nichttraumatologisch kritisch kranken Kinder mit einem C‑Problem dem Schockraum zugeführt. Dieses Ergebnis befindet sich im Einklang mit anderen Studien, die eine geringe Häufigkeit von Herz-Kreislauf-Erkrankungen im Kindes- und im Jugendalter im Gegensatz zum Erwachsenenalter im boden- und im luftgebundenen Rettungsdienst berichteten [14, 16, 17, 27]. Bei der Interpretation der Ergebnisse muss aber als Einflussfaktor das selektionierte Patientenkollektiv der vorliegenden Studie unter Ausschluss traumatologischer bzw. nichttraumatologischer Kinder ohne akute Vitalbedrohung berücksichtigt werden.

In den Reanimationsleitlinien des European Resuscitation Council (ERC) wird ausführlich auf den Herz-Kreislauf-Stillstand im Kindesalter eingegangen [27]. Rund 80 % der Patienten mit C‑Problem in der OBSERvE-DUS-PED-Studie hatten prähospital Reanimationsmaßnahmen erfahren. In anderen Studien mit unselektionierten pädiatrischen Patientenkollektiven wurden Reanimationsraten von 0,6–4 % beschrieben, diese v. a. im Säuglings-, im Kleinkind- und im jungen Schulkindalter [6, 11]. In der OBSERvE-DUS-PED-Studie lagen bei älteren Kindern (≥ 13 Jahre) vermehrt defibrillierbare Initialrhythmen vor, während bei den jüngeren Kindern (≤ 8 Jahre) die primäre Asystolie als Initialrhythmus führte. Die Daten der OBSERvE-DUS-PED-Studie stimmen mit Daten aus der Literatur überein, die im Kindesalter im Vergleich zu Erwachsenen ein respiratorisches Versagen und keine kardialen Gründe für einen Herz-Kreislauf-Stillstand im Vordergrund sehen. Auch die pädiatrischen ERC-Reanimationsleitlinien beschreiben in 40–50 % ein primär respiratorisches Problem bei Kindern als Ursache für einen Herz-Kreislauf-Stillstand [27]. Der plötzliche Kindstod wird in 20–30 % der Fälle angegeben [27]. Herz-Kreislauf-Stillstände im Rahmen eines Traumas bestehen mit einem Anteil von 10–40 % in entsprechenden Kohorten [22, 23, 27], waren aber in der OBSERvE-DUS-PED-Studie ausgeschlossen.

### Prähospitale und innerklinische Notfallmaßnahmen.

Hinweisend auf eine hohe Erkrankungsschwere der Kinder aller Altersgruppen im Studienzeitraum ist der Anteil des prähospital durchgeführten Atemwegsmanagements über 35 %. Andere Studien, in der keine spezifische pädiatrische Patientengruppe betrachtet wurde, wiesen einen Anteil an prähospitalen Atemwegssicherungen von 1,5–2,1 % für den bodengebundenen Rettungsdienst und 17,2 % für die Luftrettung nach [6, 11]. Die prähospitale Intubationsrate erwachsener kritisch kranker Patienten lag in einem Vergleichskollektiv bei nur 20 % [18]. Interessanterweise wiesen alle Kinder in der OBSERvE-DUS-PED-Studie mit prähospitaler endotrachealer Intubation einen GCS ≤ 8 infolge von D‑ und C‑Problemen (z. B. Herz-Kreislauf-Stillstand, Status epilepticus) auf. Eine aktuelle interdisziplinäre Stellungnahme zur prähospitalen Atemwegssicherung in der Kindernotfallmedizin favorisiert die Larynxmaske zur Sicherstellung einer Oxygenierung und Ventilation, da eine endotracheale Intubation nicht erzwungen werden muss [33]. In dem Patientenkollektiv der OBSERvE-DUS-PED-Studie fanden supraglottische Atemwegshilfsmittel weder prähospitale noch innerklinische Anwendung, gleichzeitig wurde keine Fehlintubation während des Studienzeitraums festgestellt. In anderen Studien mit einer ähnlichen pädiatrischen Kohorte wurden keine Umintubationen nach prähospitaler Behandlung berichtet [21], wohingegen dies in der vorliegenden Untersuchung in 16 % notwendig wurde. Zusätzlich wurden 12 % der bei Aufnahme noch nicht atemwegsgesicherten Kinder einer endotrachealen Intubation im Schockraum unterzogen. Gerade ein multiprofessionelles Team mit anästhesiologischer Beteiligung und hoher Kompetenz im Atemwegsmanagement ermöglicht die optimale Versorgung der kritisch kranken nichttraumatologischen Kinder. Zum Ende der Schockraumversorgung waren fast 60 % aller Kinder nichtinvasiv oder invasiv beatmet, dies spricht für die hohe Erkrankungsschwere des untersuchten Patientenkollektivs.

Der Anteil von rund 15 % aller kritisch kranken Kinder, die bei Schockraumaufnahme einen systolischen Blutdruck unter 80 mm Hg aufwiesen, war vergleichbar mit einer anderen Studie [21]. Während im Studienzeitraum bei kritisch kranken Kindern häufiger als in Vergleichsstudien arterielle Blutdruckmessungen bzw. zentrale Venenzugänge etabliert wurden [21], ist die Häufigkeit geringer als in Erwachsenenkollektiven am gleichen Standort [18]. Der Unterschied der Instrumentierungsrate im Vergleich zu anderen pädiatrischen Kollektiven war erwartbar und gut durch die hohe Erkrankungsschwere zu erklären.

Im Vergleich mit einem anderen Patientenkollektiv an einer Schweizer Universitätsklinik [21] wurden in der OBSERvE-DUS-PED-Studie bildgebende Verfahren (z. B. abdominelle Sonographie, Computertomographie) häufiger durchgeführt. Das lokale Versorgungskonzept für kritisch kranke nichttraumatologische Kinder sieht nach interdisziplinärer Absprache bei einer persistierenden Vigilanzminderung (GCS ≤ 8) unklarer Ätiologie eine unverzügliche Großgerätediagnostik vor. Damit spielt eine Bildgebung mittels Großgeräten und insbesondere mit dem CT bei Erwachsenen und Kindern in über 55 % der Fälle eine wichtige diagnostische Rolle [18] und bestätigt die Notwendigkeit einer räumlich nahen Lokalisation der CT zum Schockraum der Notaufnahme [13]. Hierbei kommen führend kraniale Computertomographien nach stattgehabten Reanimationsmaßnahmen (*n* = 4), hypoxischen Ereignissen (*n* = 2) und bei persistierender Vigilanzminderung zum Einsatz (*n* = 14). Eine CT anderer Körperregionen (Halswirbelsäule (*n* = 4), Thorax (*n* = 3), Abdomen (*n* = 2)) wurde selten durchgeführt. Aus zeitlichen Gründen kam ein MRT nur zu einem geringen Teil zum Einsatz.

### Verlegung nach Schockraumversorgung.

Rund 70 % der Kinder in der OBSERvE-DUS-PED-Studie wurden nach Abschluss der Schockraumversorgung auf eine Intensivstation verlegt oder direkt operativ versorgt. Die Aufnahmerate auf eine pädiatrische Intensivstation war damit deutlich höher als in einer Untersuchung mit nur 41 % [21]. Die Aufnahmerate auf eine pädiatrische Intensivstation in einer weiteren Studie mit 1,7 % erklärt sich dadurch, dass hier das Gesamtkollektiv durch den Rettungs- und Notarztdienst dem Krankenhaus zugeführter Kinder betrachtet wurde [31]. In rund 30 % der Fälle war im Einklang mit der Literatur für erwachsene Schockraumpatienten [34] nach initial erfolgter Stabilisierung im Schockraum eine Verlegung auf die Intensivstation aus medizinischen Gründen nicht mehr notwendig, und die weitere Behandlung erfolgte in der Kindernotaufnahme und auf pädiatrischen Normalstationen.

### Behandlungsergebnis.

Die 24-h-Letalität in der OBSERvE-DUS-PED-Studie betrug 11,5 % und die 30-Tages-Letalität 17,3 %. Im Vergleich zeigte ein Schweizer Kinderkollektiv eine ähnliche Mortalitätsrate von 7,2 %, wobei auch in dieser Untersuchung der überwiegende Teil der Kinder innerhalb der ersten 24–48 h verstarb. Auch die 30-Tages-Letalität von kritisch kranken nichttraumatologischen Erwachsenen am eigenen Standort war mit 18,5 % vergleichbar [18]. Hingegen war die 30-Tages-Letalität bei Kindern ohne einen prähospitalen Herz-Kreislauf-Stillstand im Untersuchungszeitraum mit 5 % deutlich niedriger als die von Erwachsenen am gleichen Standort mit 24,0 % [18].

Reanimationsereignisse sind bei Kindern im außerklinischen Setting ein seltenes Ereignis. Die 30-Tages-Überlebensrate prähospital reanimierter Kinder betrug 37 % und war damit höher als bei Erwachsenen mit 27 % [18]. Andere Studien beschreiben Überlebensraten für Kinder mit außerklinischem Herz-Kreislauf-Stillstand zwischen 5–10 % [27]. Das Deutsche Reanimationsregister wies insgesamt eine 30-Tages-Überlebensrate von 24 % bei Kindern nach, aber weniger als ein Viertel der überlebenden Kinder hat ein gutes neurologisches Behandlungsergebnis [35]. Studien belegen beim Vorliegen von initial schockbaren Rhythmen weitaus bessere Behandlungsergebnisse mit einem Überleben in bis zu 50 % der Fälle [27]. Säuglinge machen 23–50 % aller Herz-Kreislauf-Stillstände im Kindesalter im außerklinischen Setting aus, und die Prognose ist aufgrund der respiratorischen Genese häufig schlechter als bei älteren Kindern [27, 35]. Der überwiegende Anteil der Kinder im Untersuchungszeitraum verstarb im Rahmen eines C‑Problems infolge eines prähospitalen Herz-Kreislauf-Stillstandes. Alle Patienten mit primärer Asystolie verstarben, wohingegen ein Patient mit defibrillierbarem Rhythmus überlebte.

### Limitationen.

Das Studiendesign der OBSERvE-DUS-PED-Studie war eine retrospektive, monozentrische Kohortenstudie mit entsprechenden Limitationen. Hierbei wurde erstmals das Schockraummanagement für ein nichttraumatologisches Kinderkollektiv in einer zentralen Notaufnahme über einen Zeitraum von 3 Jahren untersucht. Einschränkend ist die aufgrund der selektionierenden Distributionsstrategie geringe Studiengröße von 52 Kindern mit hoher Erkrankungsschwere zu berücksichtigen. Perspektivisch bietet sich ein multizentrisches Studiendesign an, um eine größere Fallzahl zu erfassen und die in der vorliegenden Studie bereits erhobenen Ergebnisse zu verifizieren.

### Ausblick.

Zunehmende Versorgungsengpässe und fehlende Behandlungskapazitäten auf Kinderintensivstationen (z. B. infolge Personalmangels) und in Kindernotaufnahmen (z. B. infolge von „Crowding“) können zu Abweisungen kritisch kranker Kinder sowie Abmeldungen von Kinderkliniken von der Notfallversorgung führen [2, 20, 32]. Auch wenn in der OBSERvE-DUS-PED-Studie am eigenen Standort diese fehlenden intensivmedizinischen Aufnahmekapazitäten bzw. Rückmeldungen aus Maximalversorgern mit überregionalem Versorgungsauftrag nicht bestätigt werden konnten, müssen zukunftweisende Konzepte zur Kompensation und zur Sicherstellung der Versorgung kritisch kranker Kinder entwickelt werden. Hierbei erscheint es sinnvoll, die Ressource „Schockraum“ einer zentralen Notaufnahme zu nutzen: Für nichttraumatologisch kritisch kranke bzw. polytraumatisierte erwachsene Patienten liegen Weißbücher und definierte Handlungsempfehlungen für den Schockraum in zentralen Notaufnahmen vor [13]. Ebenso existieren korrespondierende Leitlinien für die Schockraumversorgung des polytraumatisierten Kindes [1, 8]. Zur Versorgung kritisch kranker nichttraumatologischer Kinder existieren für die Aufnahmelokalisation Krankenhaus, insbesondere Schockraum von Notaufnahmen bisher weder Daten noch Empfehlungen. Die OBSERvE-DUS-PED-Studie zeigt die besonderen Herausforderungen dieses vulnerablen Patientenkollektivs im Schockraum einer zentralen Notaufnahme. Aus Vulnerabilität und hohem Schadenspotenzial bei nichtzeitgerechter Behandlung leitet sich der dringende Bedarf strukturierter Leitlinien für das innerklinische Management dieser Kinder ab. Ausgehend von den Ergebnissen der vorliegenden Studie könnten zukünftige multizentrische Studien den Bedarf an materiellen, personellen und infrastrukturellen Ressourcen noch besser systematisch abschätzen und so die Grundlage für eine adäquate und zeitgerechte Behandlung liefern.

## Fazit für die Praxis

- Bisher gibt es keine Leitlinien zur materiellen oder zur personellen Ausstattung oder spezifische Handlungsempfehlungen für die Aufnahmelokalisationen und das Schockraummanagement kritisch kranker Kinder in Deutschland.
- Ziele müssen der interprofessionelle und multidisziplinäre Austausch und die Entwicklung gemeinsamer Handlungsempfehlungen und Standards sein, auch um eine „gemeinsame Sprache“ der Schockraumversorgung zu fördern.
- Klare Dispositionsstrategien, inkl. Kriterien zur Schockraumalarmierung für kritisch kranke nichttraumatologische Kinder, können personelle und technische Ressourcen in einem multidisziplinären Schockraumteam bündeln.
- Das Konzept bereits bestehender Schockraumstrukturen und die höchstmögliche fachliche Expertise in einem multidisziplinären Team (Fachexpertise) sowie einer zeitnahen Großgerätebildgebung im Schockraum von Notaufnahmen zu nutzen, erscheint sinnvoll.

## Supplementary Information

ABCDE-Probleme, Erkrankungen und Altersgruppen

## Data Availability

Die Daten werden auf begründeten Antrag zur Verfügung gestellt.
