# Supplementary material for: Retrospective analysis of the resuscitation room management of nontraumatic critically ill children in a university emergency department (OBSERvE-DUS-PED study)
Source: Anaesthesiologie. 2024 Sep 2;73(10):656–67. [Article in German] doi: 10.1007/s00101-024-01457-7 (PMC11447138; doi:10.1007/s00101-024-01457-7)
Supplement: Supplementary file 1 — ABCDE-Probleme, Erkrankungen und Altersgruppen [file 101_2024_1457_MOESM1_ESM.pdf]

Supplemental Material – Abbildungen

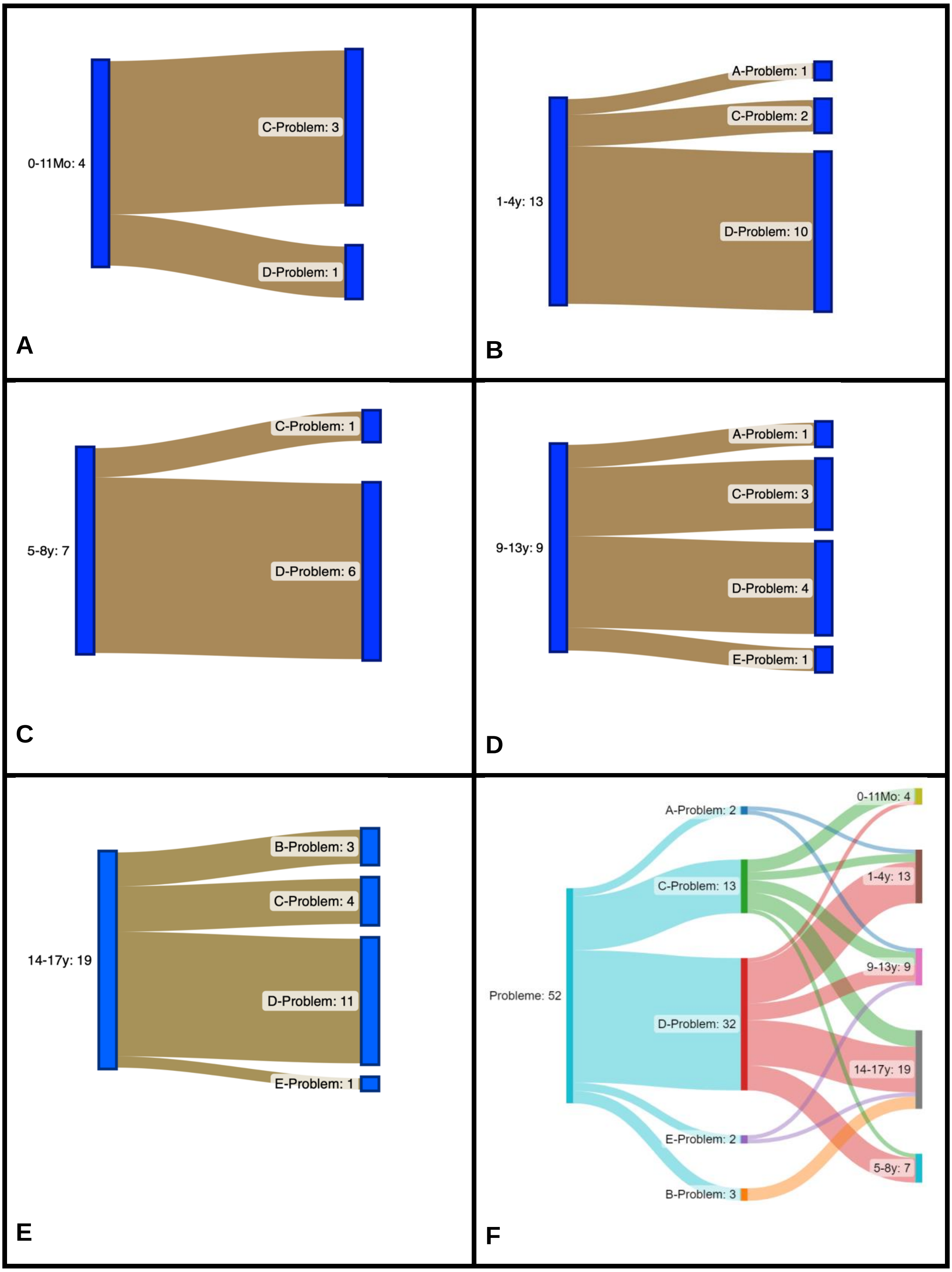

Abb.S1 Verteilung der ABCDE-Probleme in den jeweiligen Altersgruppen (A-E) und des Gesamtkollektives (F) der OBSERvE-DUS-PED-Studie (n=52)

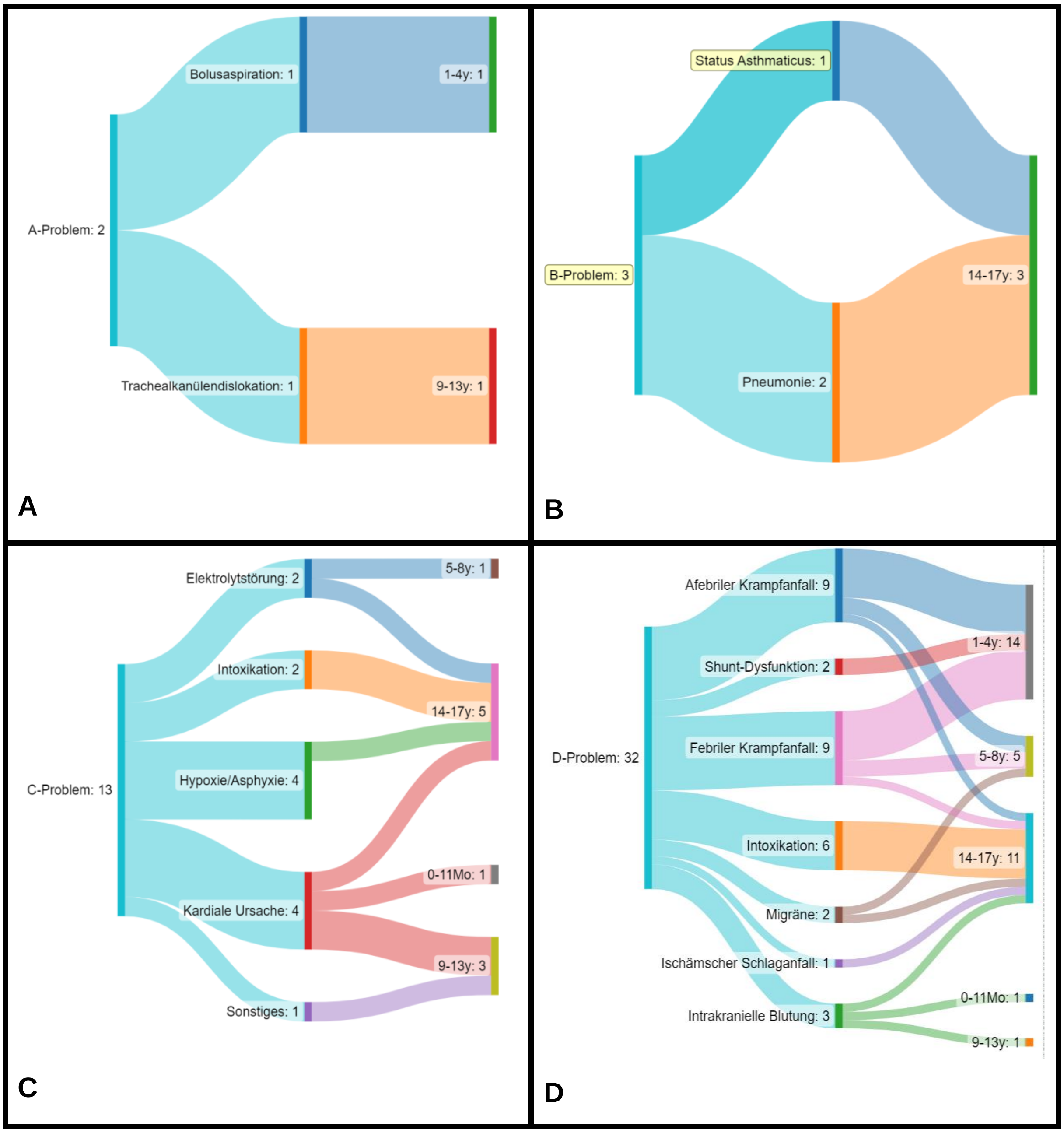

Abb.S2 Verteilung der den ABCD-Problemen zugrundeliegenden Erkrankungen und den jeweiligen Altersgruppen (E mit n=2 nicht dargestellt, beides Intoxikationen) der OBSERvE-DUS-PED-Studie (n=52)

Supplemental Material - Tabelle

**Tab.S1** Kategorisierung der prähospitalen Erkrankung- bzw. Verletzungsschwere nach dem National Advisory Committee of Aeuronautics (NACA)-Score (mod. nach [15])	
Score	Bedeutung
I	Geringfügige Störung
II	Ambulante Störung
III	Stationäre Behandlung
IV	Akute Lebensgefahr nicht auszuschließen
V	Akute Lebenbedrohung
VI	Primär erfolgreiche Reanimation
VII	Tod
